# Diversity and toxigenicity of fungi and description of *Fusarium madaense* sp. nov. from cereals, legumes and soils in north-central Nigeria

**DOI:** 10.3897/mycokeys.67.52716

**Published:** 2020-06-08

**Authors:** Chibundu N. Ezekiel, Bart Kraak, Marcelo Sandoval-Denis, Michael Sulyok, Oluwawapelumi A. Oyedele, Kolawole I. Ayeni, Oluwadamilola M. Makinde, Oluwatosin M. Akinyemi, Rudolf Krska, Pedro W. Crous, Jos Houbraken

**Affiliations:** 1 Department of Microbiology, Babcock University, Ilishan Remo, Ogun State, Nigeria Babcock University Ilishan Remo Nigeria; 2 Institute of Bioanalytics and Agro-Metabolomics, Department of Agrobiotechnology (IFA-Tulln), University of Natural Resources and Life Sciences Vienna (BOKU), Konrad Lorenzstr. 20, A-3430 Tulln, Austria University of Natural Resources and Life Sciences Vienna Tulln Austria; 3 Westerdijk Fungal Biodiversity Institute, Uppsalalaan 8, 3584 CT Utrecht, The Netherlands Westerdijk Fungal Biodiversity Institute Utrecht Netherlands; 4 Institute for Global Food Security, School of Biological Sciences, Queen’s University Belfast, University Road, Belfast, BT7 1NN, Northern Ireland, UK Queen’s University Belfast Belfast United Kingdom

**Keywords:** Aflatoxins, chemotaxonomy, food safety, *
Fusarium
*, mycology, secondary metabolites

## Abstract

Mycological investigation of various foods (mainly cowpea, groundnut, maize, rice, sorghum) and agricultural soils from two states in north-central Nigeria (Nasarawa and Niger), was conducted in order to understand the role of filamentous fungi in food contamination and public health. A total of 839 fungal isolates were recovered from 84% of the 250 food and all 30 soil samples. Preliminary identifications were made, based on macro- and micromorphological characters. Representative strains (n = 121) were studied in detail using morphology and DNA sequencing, involving genera/species-specific markers, while extrolite profiles using LC-MS/MS were obtained for a selection of strains. The representative strains grouped in seven genera (*Aspergillus*, *Fusarium*, *Macrophomina*, *Meyerozyma*, *Neocosmospora*, *Neotestudina* and *Phoma*). Amongst the 21 species that were isolated during this study was one novel species belonging to the *Fusarium fujikuroi* species complex, *F. madaense***sp. nov.**, obtained from groundnut and sorghum in Nasarawa state. The examined strains produced diverse extrolites, including several uncommon compounds: averantinmethylether in *A. aflatoxiformans*; aspergillimide in *A. flavus*; heptelidic acid in *A. austwickii*; desoxypaxillin, kotanin A and paspalitrems (A and B) in *A. aflatoxiformans*, *A. austwickii* and *A. cerealis*; aurasperon C, dimethylsulochrin, fellutanine A, methylorsellinic acid, nigragillin and pyrophen in *A. brunneoviolaceus*; cyclosporins (A, B, C and H) in *A. niger*; methylorsellinic acid, pyrophen and secalonic acid in *A. piperis*; aspulvinone E, fonsecin, kojic acid, kotanin A, malformin C, pyranonigrin and pyrophen in *A. vadensis*; and all compounds in *F. madaense* sp. nov., *Meyerozyma*, *Neocosmospora* and *Neotestudina*. This study provides snapshot data for prediction of food contamination and fungal biodiversity exploitation.

## Introduction

Fungi are ubiquitous and diverse, inhabiting various environments including agricultural soils and the crops grown on them (Stajich et al. 2009). Fungi in soil can contaminate, invade and colonise crops on the field during pre-harvest stages and can remain present during the post-harvest processing stages. Depending on the processing steps, these fungi may later spoil foods during storage or in households or markets when storage conditions are sub-optimal and climatic conditions are favourable for their growth (Prange et al. 2005, Taniwaki et al. 2018). Thus, fungal contamination and colonisation of crops could directly lead to pre- and post-harvest food losses, mycotoxin contamination and indirectly to public health risks from consumption of mycotoxin-contaminated foods (Avery et al. 2019). Additionally, soil could serve as a reservoir for pathogenic fungi, constituting public health hazards to farmers who spend much of their time on farms and have direct contact with agricultural soils. On the positive side, beneficial fungi, including biological control strains and species of industrial relevance, are also present in agricultural soils, waiting to be explored (Donner et al. 2009, Bautista-Rosales et al. 2013, Bandyopadhyay et al. 2016).

Proper characterisation of fungi is fundamental to effectively determine their ecology and roles in the environment. In Nigeria, several studies have focused on fungal contamination of food crops (Adebajo et al. 1994, Bankole et al. 2003, Marley et al. 2004, Afolabi et al. 2006, Adejumo et al. 2007, Atehnkeng et al. 2008, Makun et al. 2009, 2011, Fapohunda et al. 2012, Abdus-Salaam et al. 2016, Oyedele et al. 2017, Ezekiel et al. 2013a, 2013b, 2014, 2016, 2019, Frisvad et al. 2019, Akinfala et al. 2020) and soil (Donner et al. 2009, Bandyopadhyay et al. 2019, Ezekiel et al. 2019). Many of these reports focused mainly on characterising aflatoxigenic *Aspergillus* species, because of their high incidence and their ability to produce aflatoxins and less on other mycotoxins produced by other fungal genera and species. Thus, studies on characterisation of other fungi including *Fusarium*, a genus also comprising important mycotoxin producers, have rarely been conducted in Nigeria (Marley et al. 2004, Afolabi et al. 2006, Adejumo et al. 2007, Makun et al. 2011, Fapohunda et al. 2012). Regardless of the fungal genera studied, the application of robust taxonomic tools comprising a combination of phenotypic characterisation, DNA sequence-based methods and extrolite profiling for fungal identification is scarce (Frisvad et al. 2019, Ezekiel et al. 2020, Akinfala et al. 2020). This comprehensive approach is valuable due to the high precision, based on the use of species-specific DNA markers (Houbraken et al. 2011, 2012, Samson et al. 2014, 2019).

Therefore, in view of the need to understand the roles of fungi in food contamination and other processes, we conducted a mycological investigation into agricultural crops (foods) commonly consumed and available in agrarian households and soils on which the crops were grown in two north-central states (Nasarawa and Niger) in Nigeria. The two states were selected for this study, based on previous reports (Adetunji et al. 2014, Abdus-Salaam et al. 2015, Oyedele et al. 2017) that implicated these states and/or the agro-ecological zone to which they belong (Southern Guinea Savanna) as regions of moderate-to-high aflatoxin and fumonisin contamination in foods. Consequently, it was necessary to study the fungal diversity in these states.

## Materials and methods

### Food and soil sampling

Various food (n = 250) and soil (n = 30) samples were collected in two states (Nasarawa and Niger) in north-central Nigeria. Samples were collected in September 2018 (harvest season) and January 2019 (storage season). The distribution of samples by sampling season were: harvest (food, n = 143) and storage (food, n = 107; soil, n = 30). Samples were collected from households within one week of harvest and after three months of storage (storage samples). In each state, food samples (1 kg per sample) were collected from households within three randomly selected communities that are at least 5–20 km apart: Mada station, Tundun Adabu and Yelwa Doma in Nasarawa state and Diko, Nubwa Koro and Sabon Wuse in Niger state. The food samples collected included: cowpea (n = 7); groundnut (n = 53), maize (n = 142), millet (n = 1), rice (n = 23) and sorghum (n = 24). Soil samples were collected from the farmlands belonging to five randomly selected households in each community. Sampled fields were at least 1 km apart. In each field, one composite sample (90–100 g) was collected by traversing the field and taking five subsamples from random points. The depth of soil sampling was 3–4 cm.

Food samples were placed in polyethylene bags whilst soil samples were placed in paper bags. All food samples were fragmented in an electric blender (MX-AC400, Panasonic, India) and stored at 4°C prior to analysis within 48 h. Soil samples were transferred to plastic bags and clods were crushed using a mortar and pestle. Soil samples were then homogenised by hand-mixing prior to immediate fungal analysis.

### Mycological studies of food and soil

Fungal isolation

Filamentous fungi, present in the food and soil samples, were isolated and enumerated using the dilution plating technique described by Samson et al. (1995). The fragmented samples (10 g each) were diluted in sterile distilled water (90 ml). Each mixture was homogenised on a vortex mixer for 2 min prior to surface-plating of 100 µl on malt extract agar (MEA; Oxoid, UK). The inoculated plates were incubated at 25 °C for 3 to 5 d. The number of fungal colonies on the plates was counted and the colony forming units per gram (CFU/g) of the analysed samples calculated. Distinct colonies, appearing on the isolation plates, were carefully transferred to freshly prepared MEA plates and incubated at 25 °C for 7 d. All pure cultures were stored at 25 °C on MEA slants in 4 ml vials covered with sterile distilled water.

Characterisation of fungal isolates

Fungal isolates from the food and soil samples were characterised, based on morphological characteristics, DNA sequence data and/or secondary metabolites. The strains were first cultivated on MEA and assessed for macro- and microscopic characters, which were then compared with descriptions in appropriate keys (Frisvad and Samson 2004, Leslie and Summerell 2006, Pitt and Hocking 2009, Samson et al. 2011, 2019). Phenotypically similar isolates were grouped and selected isolates representing each group were identified using a sequence-based approach. For *Fusarium* and *Neocosmospora* spp., colony features and growth rates were assessed using MEA, oatmeal agar (OA), potato dextrose agar (PDA; recipes in Crous et al. 2019) and synthetic nutrient-poor agar (SNA; Nirenberg 1976); and micromorphology was studied using carnation leaf agar (CLA; Fisher et al. 1982) and SNA following protocols described elsewhere (Leslie and Summerell 2006, Sandoval-Denis et al. 2018). For the molecular analysis, DNA was extracted from each selected isolate previously cultivated on MEA at 25 °C for 5 d. Parts of the β-tubulin (*BenA*) and calmodulin (*CaM*) genes of the *Aspergillus* isolates were amplified and sequenced as previously described (Houbraken et al. 2011, 2012, Samson et al. 2019). The ITS regions, a part of the translation elongation factor 1 alpha (*TEF-1α*) and/or the RNA polymerase II second largest subunit (*RPB2*) gene of all the other fungal species were amplified and sequenced in accordance with Groenewald et al. (2005), Groenewald et al. (2013), Chen et al. (2015), Chen et al. (2017) and O’Donnell et al. (1998, 2010). Additionally, partial fragments of the *BenA*, *CaM*, *TEF-1a* and RNA polymerase II largest subunit (*RPB1*) were generated for a subset of *Fusarium* strains, according to O’Donnell et al. (1998, 2009, 2010) and Woudenberg et al. (2009). All generated sequences were compared with the sequences present in the NCBI database and internal curated databases of the Westerdijk Fungal Biodiversity Institute (WI) for confirmation of species identities. The identified isolates are maintained in the working culture collection of WI (“DTO culture collection”) and in the culture collection of WI (“CBS culture collection”). All newly-generated sequences are deposited in GenBank (Suppl. material 1: Table S1).

To further explore the species diversity and determine the presence of putative novel taxa amongst the fusaria, phylogenetic analyses were carried out, based on *BenA, CaM, RPB1, RPB2* and *TEF-1a* sequences. A first analysis, based on partial *RPB2* sequences, was intended to determine the generic distribution of the Nigerian isolates. A second multi-locus analysis, based on the five gene regions above-mentioned, was used to determine the genetic exclusivity of an undescribed phylogenetic clade belonging to the *Fusarium fujikuroi* species complex (FFSC, O’Donnell et al. 2015, Choi et al. 2018). Additional sequences of type and reference strains were retrieved from GenBank and included in the analyses (Suppl. material 1: Table S2). Sequences of the individual loci were aligned using MAFFT v. 7.110 (Katoh et al. 2017). The individual gene datasets were assessed for incongruency prior to concatenation using a 70% reciprocal bootstrap criterion (Mason-Gamer and Kellogg 1996). Phylogenetic analyses were based on Maximum-Likelihood (ML) and Maximum-Parsimony (MP). For ML, randomised accelerated (sic) ML (RAxML) for high performance computing (Stamatakis 2014) was used on the CIPRES Science Gateway portal (Miller et al. 2012) and clade stability was tested with a bootstrap analysis (BS) using default parameters. For MP, PAUP (Phylogenetic Analysis Using Parsimony, v. 4.0b10; Swofford 2003) was used and phylogenetic relationships were estimated by heuristic searches with 1000 random addition sequences with tree-bisection-reconnection and branch swapping option set to ‘best trees’ only. All characters were weighted equally and alignment gaps treated as missing data. Tree length (TL), consistency index (CI), retention index (RI) and homoplasy index (HI) were calculated. Clade stability was assessed by bootstrap analyses, based on 1000 replications.

For extrolite profiling, each representative *Aspergillus* isolate was grown on Czapek yeast autolysate (CYA) agar, MEA and yeast extract sucrose (YES) agar and the selected *Fusarium* and *Neocosmospora* strains were grown on OA and PDA prior to extraction (Yilmaz et al. 2014, Samson et al. 2019). The culture media were incubated for 7 and 14 d at 25 °C. The agar plug extraction method of Filtenborg et al. (1983) with modifications as described by Smedsgaard (1997) was applied to extract cultural compounds. Extraction solvent for the agar plugs included ethylacetate/dichloromethane/methanol (3:2:1, v/v/v) containing 1% formic acid. All extracts were air-dried prior to LC–MS/MS screening as described below.

### LC-MS/MS extrolite analysis of agar plug extracts

Extrolites of fungal cultures were determined by a dilute and shoot LC–MS/MS method (Sulyok et al. 2020). The air-dried extracts were dissolved in 1 ml (ratio 1:1, v/v) of extraction solvent (acetonitrile/water/acetic acid 79:20:1, v/v/v) and then diluted with acetonitrile/water/acetic acid 20:79:1, v/v/v in a 1:1 (v/v) ratio prior to injection into the LC–MS/MS instrument. The QTrap 5500 LC–MS/MS System (Applied Biosystems, Foster City, CA, USA), equipped with TurboIonSpray electrospray ionisation (ESI) source and a 1290 Series HPLC System (Agilent, Waldbronn, Germany) was applied to screen the compounds. Chromatographic separation was performed at 25 °C on a Gemini C18–column, 150 × 4.6 mm i.d., 5 μm particle size, equipped with a C18 4 × 3 mm i.d. security guard cartridge (Phenomenex, Torrance, CA, USA). The chromatographic method, chromatographic and mass spectrometric parameters are as described by Sulyok et al. (2020). ESI-MS/MS was conducted in the time-scheduled multiple reaction monitoring (MRM) mode both in positive and negative polarities in two separate chromatographic runs per sample by scanning two fragmentation reactions per analyte. The MRM detection window of each analyte was set to its expected retention time ± 20 s and ± 26 s in the positive and the negative modes, respectively. The identified positive analytes were confirmed by the acquisition of two MRMs per analyte. This yielded 4.0 identification points, according to European Commission decision 2002/657 (EC 2002). Additionally, the LC retention time and the intensity ratio of the two MRM transitions were in agreement with the related values of an authentic standard within 0.03 min and 30%, respectively, following the criteria for mycotoxin identification as laid down in SANTE 12089/2016.

### Data analysis

The IBM SPSS v21.0 (SPSS Inc., IL, USA) was applied for data analysis. Data on fungal load were first normalised by a logarithm to base 10 transformation of the original data prior to the calculation of mean values. Means were tested for significance by One-way ANOVA (α = 0.05). Means of the concentrations (µg/kg) of the extrolites, produced by the fungal strains in culture media, were also calculated.

## Results and discussion

### Distribution of fungi in food and soil

**Figure 1.**
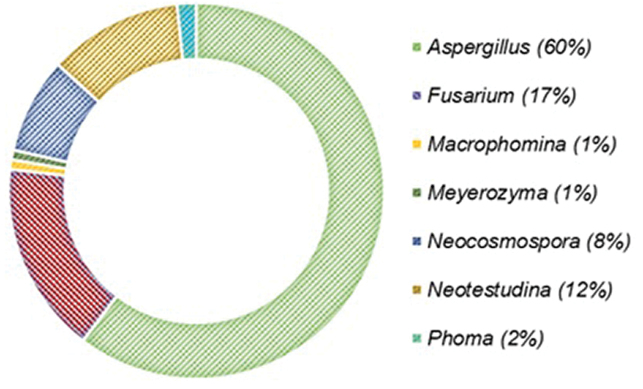
Overall incidence of fungal genera recovered from food and soil in two states in north-central Nigeria.

**Figure 2.**
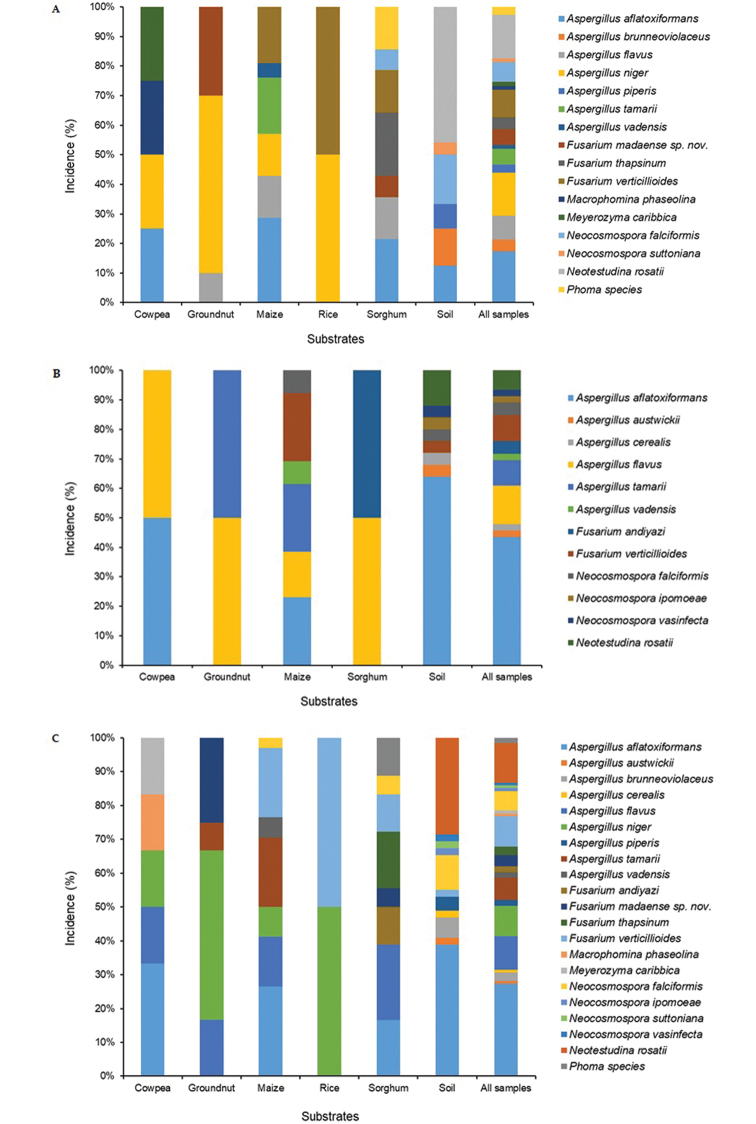
Distribution of fungal species in food and soil in two states **A** Nasarawa state **B** Niger state **C** combined/both states) in north-central Nigeria.

Fungal propagules were recovered from 84% (n = 209) of the 250 food samples and from all of the soil samples (n = 30). The fungal load in the food samples was significantly (*p* < 0.05) higher at harvest (range: 2.00–6.22; mean: 4.07 ± 0.95 Log_10_CFU/g) than in storage (range: 2.00–4.60; mean: 3.44 ± 0.69 Log_10_CFU/g). The load of fungal propagules in the soil samples ranged 2.70–4.20 (mean: 3.45 ± 0.34 Log_10_CFU/g). Variations observed in fungal load during the two seasons (harvest and storage) may be attributed to the sampling environment and nature of samples. For example, harvest samples were recently collected from the field where crops are in contact with soil and a large diversity of fungal propagules were present (Bankole et al. 2006), whereas storage conditions are often controlled (crops stored individually in local granaries), thereby leading to lower fungal densities (Williams et al. 2014). In addition, harvest samples were not yet “cleaned” (threshed or deshelled) and so harboured more viable fungal propagules compared to samples collected from storage bins which were already threshed, deshelled and bagged. A similar fungal load found in soil samples in the present study was previously reported for 55 soil samples collected from maize fields (range: 55–3736 CFU/g = 1.74–3.57 Log_10_CFU/g) across three agro-ecological zones of Nigeria (Donner et al. 2009). A total of 839 fungal isolates were recovered from the food and soil samples and grouped, based on similarities in phenotypic characters. Representative isolates (n = 121) selected from the groups clustered into seven genera (Fig. 1) and 21 species (Fig. 2C), based on a polyphasic taxonomic scheme. The overall incidences of the recovered fungal genera in decreasing order of magnitude were: *Aspergillus* (60%), *Fusarium* (17%), *Neotestudina* (12%), *Neocosmospora* (8%), *Phoma* (2%), *Macrophomina* (1%) and *Meyerozyma* (1%). The overall highest incidence of *Aspergillus* in the samples, as recorded in the present study, agrees with previous studies from different locations and substrates (Atehnkeng et al. 2008, Donner et al. 2009, Diedhiou et al. 2011, Makun et al. 2011, Ezekiel et al. 2013a, 2013b, 2016, 2019, Probst et al. 2014, Oyedele et al. 2017). To the best of our knowledge, we present the first report of *Neotestudina* from Nigerian soil.

Based on the fungal isolates recovered from food and soil samples and identified in this study, sample type-specific fungal incidences were estimated as 40.5%, 28%, 14.9%, 9.9%, 4.9% and 1.7% in soil, maize, sorghum, groundnut, cowpea and rice, respectively. Aspergilli were widely distributed in soil and food, although a higher proportion of isolates (35.6%) was recovered from soil compared to the individual foods. Nine *Aspergillus* species, belonging to two sections, were recovered in this study (Fig. 2). The species include: *A. aflatoxiformans*, *A. austwickii*, *A. cerealis*, *A. flavus* and *A. tamarii* in section Flavi (Frisvad et al. 2019) and *A. brunneoviolaceus*, *A. niger*, *A. piperis* and *A. vadensis* in section Nigri (Samson et al. 2007, Perrone et al. 2011, Varga et al. 2011). *Aspergillus aflatoxiformans* (27.3%) was the predominant species found in this study, based on the identified representative isolates (Fig. 2C). However, the predominance of *A. aflatoxiformans* here contradicts several previous reports that presented *A. flavus* as the predominant *Aspergillus* species in food and crops in Nigeria and elsewhere (Atehnkeng et al. 2008, Donner et al. 2009, Ezekiel et al. 2013a, 2013b, 2016, 2019, Probst et al. 2014, Oyedele et al. 2017). The disparity between our finding and previous reports is explainable and owes to bias during sub-culturing and selection of fungal isolates for molecular identification as less emphasis was given to *A. flavus* isolates. With respect to location, *A. austwickii* and *A. cerealis* were recovered only from soil in Niger state, while *A. brunneoviolaceus*, *A. niger* and *A. piperis* were recovered only in food and soil from Nasarawa state. Here, we present for the first time, *A. vadensis* as isolates from Nigerian food.

Four *Fusarium* species (*F. andiyazi*, *F. madaense* sp. nov., *F. thapsinum* and *F. verticillioides*), belonging to FFSC, were identified. All the *Fusarium* spp. were recovered from food samples, except *F. verticillioides* that was found in both food (maize, rice and sorghum) and soil (Fig. 2C). *Fusarium andiyazi* was isolated from sorghum in the Nasarawa state (Fig. 2A), *F. andiyazi* was specific to sorghum from Niger state (Fig. 2B) and *F. madaense* sp. nov. was recovered from groundnut and sorghum from Nasarawa state (Fig. 2A). The diversity of *Fusarium* observed in this study is remarkable, as we present unique occurrences of *F. madaense* sp. nov. and *F. thapsinum* from Nigerian grains, in addition to the previously reported *F. andiyazi* and *F. verticillioides* (Marley et al. 2004, Afolabi et al. 2006, Makun et al. 2009, 2011). *Fusarium verticillioides* and other members of the FFSC, including those found in the present study (*F. andiyazi*, *F. proliferatum*, *F. pseudonygamai*, *F. subglutinans* and *F. thapsinum*), have been documented in cereals and natural environments in different countries (Fandohan et al. 2005, Ncube et al. 2011, Leyva-Madrigal et al. 2014, O’Donnell et al. 2015, Moussa et al. 2017, Choi et al. 2018, Chala et al. 2019). The specificity of *F. andiyazi* and *F. thapsinum* to sorghum, which we observed here, agrees with literature (Marley et al. 2004, Pena et al. 2018, Chala et al. 2019). The additional discovery of *F. madaense* sp. nov. in groundnut and sorghum in this study emphasises the need to adopt adequate and robust characterisation approaches in fungal studies, as well as to conduct large-scale fungal biodiversity studies of food and soil in the country.

*Macrophomina phaseolina*, *Meyerozyma caribbica* and *Phoma* species were isolated only from food in Nasarawa state (Fig. 2A, C). *Macrophomina phaseolina* and *M. caribbica* were specific to cowpea, while *Phoma* species were recovered from sorghum. *Macrophomina phaseolina* is a common pathogen of legumes, including cowpea and causes charcoal rot and root rot (Amusa et al. 2007, Oladimeji et al. 2012, Sarr et al. 2014, Khan et al. 2017). *Meyerozyma caribbica* (anamorph *Candida fermentati*) is a halophilic and rhizospheric yeast with biological control potential against phytopathogenic fungi (Bautista-Rosales et al. 2013), while *Phoma* is a genus of mostly phytopathogens (Chen et al. 2015, 2017). Future studies may explore the role of *M. caribbica* in biological control of mycotoxigenic fungi found in this study. *Neotestudina rosatii* was present only in soil (Fig. 2C). This fungus was actually first described in Africa in 1961 as the agent for maduromycosis (Segretain and Destombes 1961) and has been associated with the same human disease in two African countries, Senegal and Somalia (Baylet et al. 1968, Destombes et al. 1977). Four species of *Neocosmospora* (*N. falciformis*, *N. ipomoeae*, *N. suttoniana* and *N. vasinfecta*) were recovered from food and soil (Fig. 2C). *Neocosmospora ipomoeae* and *N. vasinfecta* and *N. suttoniana* were specific only to soils in Niger and Nasarawa states, respectively, while *N. falciformis* was found in food and soil in both states (Fig. 2A, B). *Neocosmospora* (formerly ‘*Fusarium*’ solani species complex, FSSC) comprises common pathogens of plants, humans and animals (Sandoval-Denis and Crous 2018). For example, *N. falciformis* (syn. *F. falciforme*) is known to be associated with diverse cutaneous and subcutaneous fungal infections (Dignani and Anaissie 2004, Garcia et al. 2015). This species is frequently found in equine ocular infections and in canines and reptiles (O’Donnell et al. 2016). Recently, Sandoval-Denis and Crous (2018) described *N. suttoniana*; this species is implicated in uncommon human eye infections in Africa and the USA (O’Donnell et al. 2008).

### Extrolites produced in fungal cultures

The elucidation of extrolite patterns from fungal strains grown on mycological media, using the highly sensitive LC-MS/MS technique, remains the gold standard chemotaxonomic approach to fungal characterisation (Frisvad et al. 2007, 2018, Samson et al. 2019). In this study, extrolite production in solid media was examined by LC-MS/MS in strains belonging to 20 of the 21 identified fungal species. Cultures of *A. tamarii* and *M. phaseolina* were not included in extrolite analysis. Whereas all compounds were quantitatively determined, aflatrem, asparason A, aspulvinone E, aurasperons, desoxypaxillin, fonsecin, nigragillin, paspalin, paspalinin, paspalitrems and tensidol B were only semi-quantified in the cultures due to lack of a quantitative standard. All the examined fungal strains/species produced at least three (Tables 1, 2) and as many as 33 compounds in *A. aflatoxiformans* (Table 1). Brevianamid F and cyclo(L-Pro-L-Tyr) were detected in examined cultures and cyclo(L-Pro-L-Val) was present in all except three *Neocosmospora* species (*N. falciformis*, *N. ipomoeae* and *N. suttoniana*). These three compounds, found in almost all fungal species in this study, were the only metabolites detected in cultures of *M. caribbica* and *Phoma* sp. in addition to tryptophol in *M. caribbica* (data not shown). Brevianamid F, cyclo(L-Pro-L-Tyr) and cyclo(L-Pro-L-Val) were previously reported in cultures of *A. niger*, *A. tamarii*, *Paecilomyces* and *Talaromyces* from cocoa beans processing in Nigeria (Akinfala et al. 2020).

**Table 1.** Extrolite production in *Aspergillus* cultures.

Extrolites	*Aspergillus aflatoxiformans*	*Aspergillus austwickii*	*Aspergillus brunneoviolaceus*	*Aspergillus cerealis*	*Aspergillus flavus*	*Aspergillus niger*	*Aspergillus piperis*	*Aspergillus vadensis*
3-Nitropropionic acid	+	+	-	+	+	-	-	-
Aflatoxicol	+	-	-	-	+	-	-	-
Aflatoxin B_1_	+	+	-	+	+	-	-	-
Aflatoxin B_2_	+	+	-	+	+	-	-	-
Aflatoxin G_1_	+	+	-	+	-	-	-	-
Aflatoxin G_2_	+	+	-	+	-	-	-	-
Aflatoxin M_1_	+	+	-	+	+	-	-	-
Aflatrem	+	+	-	+	+	-	-	-
Asparason A	+	+	-	+	+	-	-	-
Asperfuran	+	+	-	+	+	-	-	-
Aspergillimide	-	-	+	-	+	-	-	-
Aspulvinone E	-	-	-	-	-	+	+	+
Aurasperon B	-	-	-	-	-	-	+	+
Aurasperon C	-	-	+	-	-	-	+	+
Aurasperon G	-	-	-	-	-	-	+	+
Averantin	+	+	-	+	+	-	-	-
Averantinmethylether	+	-	-	-	-	-	-	-
Averufin	+	+	-	+	+	-	-	-
Brevianamid F	+	+	+	+	+	+	+	+
Citreorosein	-	-	+	-	-	-	-	-
cyclo(L-Pro-L-Tyr)	+	+	+	+	+	+	+	+
cyclo(L-Pro-L-Val)	+	+	+	+	+	+	+	+
Cyclopiazonsäure	+	+	-	+	+	-	-	-
Cyclosporin A	-	-	-	-	+/-	+	-	-
Cyclosporin B	-	-	-	-	+/-	+	-	-
Cyclosporin C	-	-	-	-	+/-	+	-	-
Cyclosporin H	-	-	-	-	+/-	+	-	-
Demethylsulochrin	-	-	+	-	-	-	-	-
Desoxypaxillin	+	+	-	+	+	-	-	-
Emodin	-	-	+	-	-	+	-	-
Endocrocin	-	-	+	-	-	+	-	-
Fellutanine A	-	-	+	-	-	-	-	-
Fonsecin	-	-	-	-	-	+	+	+
Heptelidic acid	-	+	-	-	+	-	-	-
Iso-Rhodoptilometrin	-	-	+	-	-	+	-	-
Kojic acid	+	+	-	+	+	+	-	+
Kotanin A	+	+	-	+	+	+	-	+
Malformin A	-	-	-	-	-	+	-	-
Malformin C	-	-	-	-	-	+	+	+
Meleagrin	-	-	+	-	-	-	-	-
Methylorsellinic acid	-	-	+	-	-	-	+	-
Nidurufin	+	+	-	+	+	-	-	-
Nigragillin	-	-	+	-	-	+	+	+
Norsolorinic acid	+	+	-	+	-	-	-	-
O-Methylsterigmatocystin	+	+	-	+	+	-	-	-
Oxaline	-	-	+	-	-	-	-	-
Paraherquamide E	-	-	+	-	-	-	-	-
Paspalin	+	+	-	+	+	-	-	-
Paspalinin	+	+	-	+	+	-	-	-
Paspalitrem A	+	+	-	+	+	-	-	-
Paspalitrem B	+	+	-	+	+	-	-	-
Pyranonigrin	-	-	-	-	-	+	+	+
Pyrophen	-	-	+	-	-	+	+	+
Secalonic acid D	-	-	+	-	-	-	+	-
Sporogen AO1	-	-	-	-	+	-	-	-
Sterigmatocystin	+	+	-	+	+	-	-	-
Tensidol B	-	-	-	-	-	+	-	-
Versicolorin A	+	+	-	+	+	-	-	-
Versicolorin C	+	+	-	+	+	-	-	-
Versiconal acetate	+	+	-	+	-	-	-	-

Extrolite produced (+); Extrolite not produced (-). Produced only by the non-aflatoxigenic strain (+/-).

*Aspergillus* metabolites

The extrolite patterns of the *Aspergillus* species, isolated and identified in this study, except *A. tamarii* which was not evaluated, are shown in Table 1. Members of the section Flavi produced metabolites (aflatoxins and their biosynthetic pathway precursors, asparason A, cyclopiazonic acid, desoxypaxillin, kojic acid, kotanin A, paspalin and paspalitrems) consistent with previous reports (Frisvad et al. 2019, Uka et al. 2019, Ezekiel et al. 2020). However, some new findings are reported herein. For example, desoxypaxillin, heptelidic acid, kotanin A and paspalitrems were previously reported in *A. flavus* (Uka et al. 2019, Kovac et al. 2020, Ezekiel et al. 2020), but not in *A. aflatoxiformans*, *A. austwickii* and *A. cerealis*. Hence, we present the first report of desoxypaxillin, kotanin A (mean: 534 µg/kg) and paspalitrems in the three S-type sclerotium (minisclerotium) producing species and heptelidic acid (10.2 mg/kg) only in *A. austwickii*. In addition, averantinmethylether (9.4 µg/kg) and aspergillimide (21.3 µg/kg) are two uncommon compounds found only in cultures of one strain of *A. aflatoxiformans* and *A. flavus*, respectively. Similar to a recent report (Ezekiel et al. 2020), two of the four *A. flavus* strains, examined in this study, produced sporogen AO1 (mean: 974 µg/kg), confirming the production of this compound in *A. flavus*. One of the four *A. flavus* strains, DTO 421-G6, did not produce aflatoxins, any of its pathway metabolites, cyclopiazonic acid or kojic acid, but produced cyclosporins A, B, C and H. Cyclosporin production has been reported in *Aspergillus terreus* (Sallam et al. 2003), *Neocosmospora solani* (Sawai et al. 1981) and *Tolypocladium* (El-Enshasy et al. 2008). It is, therefore, suggested that strain DTO 421-G6, whose origin is sorghum grain from Sabon Wuse in Niger state, may be a prospective candidate for biological control of aflatoxins in view of its inability to biosynthesise aflatoxins, its pathway metabolites and cyclopiazonic acid. The biological control product, aflasafe, commercially available for aflatoxin control in Nigeria, contains strains of *A. flavus* original to Niger state and which possesses a similar inability to secrete the aforementioned metabolites (Bandyopadhyay et al. 2016, 2019).

The members of Aspergillus section Nigri (*A. brunneoviolaceus*, *A. niger*, *A. piperis* and *A. vadensis*) secreted a total of 31 extrolites (Table 1). However, only three extrolites, aurasperons, nigragillin and pyrophen, were common to all four species within this section. Varga et al. (2011) placed *A. brunneoviolaceus* in the *A. aculeatus* clade, whilst *A. niger*, *A. piperis* and *A. vadensis* were grouped into the *A. niger* clade. In the present study, members of the *A. niger* clade shared only four (aspulvinone E, fonsecin, malformins and pyranonigrin) of the compounds. Obviously, high variability in the types of metabolites produced was recorded amongst these closely-related species (Frisvad et al. 2007, Samson et al. 2007). Strains of *A. brunneoviolaceus* (syn. *A. fijiensis*) liberated several known extrolites: aspergillimide (mean: 30.1 mg/kg), emodin (mean: 541 µg/kg), endocrocin (mean: 23 mg/kg), iso-rhodoptilometrin (mean: 340 µg/kg), meleagrin (mean: 7.6 mg/kg), oxaline (mean: 17.3 mg/kg), paraherquamide E (mean: 6 mg/kg) and secalonic acid D (mean: 64.7 mg/kg) (Varga et al. 2011, Vesth et al. 2018, Ezekiel et al. 2020). However, citreorosein and tryprostatin B, two compounds recently reported to be produced by *A. brunneoviolaceus* from *garri* (farinated cassava) in Nigeria (Ezekiel et al. 2020), were not detected in cultures of the present strains. Nonetheless, six uncommon compounds (aurasperon C, dimethylsulochrin (mean: 1.9 mg/kg), fellutanine A (152 µg/kg), methylorsellinic acid (mean: 1.2 mg/kg), nigragillin and pyrophen (mean: 190 µg/kg)) were produced by strains examined in the present study. Of all the extrolites found in cultures of the four species within the section Nigri, aspergillimide, dimethylsulochrin, fellutanine A, meleagrin, oxaline and paraherquamide E were specific to only *A. brunneoviolaceus*, whilst cyclosporins (A, B, C and H) and tensidol B were unique to *A. niger*. This is the first report of cyclosporin production in *A. niger*; only 2/9 of the strains, DTO 422-H5 and DTO 422-H6, were implicated here. In addition, we observed emodin (48 µg/kg) and endocrocin (262 µg/kg) production in only one strain (DTO 421-I7) of *A. niger*. All other compounds found in cultures of *A. niger* in the present study are known extrolites (de Vries et al. 2005, Samson et al. 2007, Nielsen et al. 2009, Perrone et al. 2011, Akinfala et al. 2020).

Two strains of *A. piperis*, screened in this study, secreted compounds agreeable to those previously documented in literature (Samson et al. 2007, Ezekiel et al. 2020). Amongst the extrolites found in the present study are those being reported here for the first time in *A. piperis*: methylorsellinic acid (mean: 1.7 mg/kg), pyrophen (mean: 50 mg/kg) and secalonic acid (24.3 μg/kg) (Table 1). de Vries et al. (2005) examined extrolite production in one strain of *A. vadensis* and found aurasperon B, asperazine, nigragillin and a more polar kotanin-like compound. Here, we report aspulvinone E, aurasperons (B, C and G), fonsecin, kojic acid (mean: 281 µg/kg), kotanin A (91 µg/kg), malformin C (mean: 857 µg/kg), nigragillin, pyranonigrin (mean: 72 mg/kg) and pyrophen (mean: 52.4 mg/kg). The complexity in small molecule chemical profiles observed in the species belonging to the section Nigri suggests a high degree of close-relatedness amongst these species (Samson et al. 2007, Nielsen et al. 2009, Perrone et al. 2011).

Extrolites from *Fusarium* and its related fungal species

A total of 15, 12 and 11 extrolites were found in cultures of *Fusarium*, *Neocosmospora* and *Neotestudina* (Table 2). With the exception of *N. vasinfecta*, gibepyron D production was shared by all examined strains of these three genera; higher quantities were found in cultures of *F. madaense* sp. nov. (mean: 4.9 mg/kg) and *F. thapsinum* (mean: 4.2 mg/kg). Gibepyron D is an oxidised derivative of gibepyrone A that has been reported in *F. fujikuroi*, *F. oxysporum* and *F. proliferatum* (Wang et al. 2011, Liu et al. 2013, Janevska et al. 2016). Thus, its production by three fungal genera suggests ancestral relatedness of a gene cluster encoding production of this compound (Janevska et al. 2016). Fumonisins (FA_1_ (mean: 616 µg/kg), FB_1_ (mean: 3.4 mg/kg), FB_2_ (mean: 2 mg/kg) and FB_3_ (mean: 1.4 mg/kg)), fusarin C (mean: 27 mg/kg) and fusarinolic acid (mean: 84,856 mg/kg) were exclusively produced by the *Fusarium* species examined in this study. Fumonisins were produced as expected only by *F. verticillioides*, although the cultures of two strains, DTO 421-G2 and DTO 424-H5, did not contain any of the fumonisins. Fumonisin production is a signature in this species as well as in other selected members of the FFSC not found in the present study (Makun et al. 2011, Ncube et al. 2011, de Oliveira Rocha et al. 2011, Fanelli et al. 2012, Rocha et al. 2016, Choi et al. 2018).

**Table 2.** Extrolite production in cultures of *Fusarium* and related genera.

**Extrolites**	***Fusarium andiyazi***	***Fusarium thapsinum***	***Fusarium verticillioides***	***Fusarium madaense* sp. nov.**	***Neocosmospora falciformis***	***Neocosmospora ipomoeae***	***Neocosmospora suttoniana***	***Neocosmospora vasinfecta***	***Neotestudina rosatii***
Beauvericin	-	-	-	+	-	-	-	-	+
Bikaverin	+	+	+	+	-	-	-	-	+
Brevianamid F	+	+	+	+	+	+	+	+	+
cyclo(L-Pro-L-Tyr)	+	+	+	+	+	+	+	+	+
cyclo(L-Pro-L-Val)	+	+	+	+	-	-	-	+	+
Cyclosporin A	-	-	-	-	+	-	-	+	-
Cyclosporin B	-	-	-	-	+	-	-	+	-
Cyclosporin C	-	-	-	-	+	-	-	+	-
Cyclosporin D	-	-	-	-	+	-	-	+	-
Cyclosporin H	-	-	-	-	+	-	-	+	-
Deoxyfusapyron	+	-	-	+	-	-	-	-	+
Fumonisin A_1_	-	-	+	-	-	-	-	-	-
Fumonisin B_1_	-	-	+	-	-	-	-	-	-
Fumonisin B_2_	-	-	+	-	-	-	-	-	-
Fumonisin B_3_	-	-	+	-	-	-	-	-	-
Fusapyron	+	-	-	+	-	-	-	-	+
Fusaric acid	+	+	+	+	-	-	-	+	+
Fusarin C	-	+	+	+	-	-	-	-	-
Fusarinolic acid	+	+	+	+	-	-	-	-	-
Gibepyron D	+	+	+	+	+	+	+	+	+
Radicicol	-	-	-	-	-	-	-	+	-
Sulochrin	-	-	-	-	-	-	-	-	+
Tryptophol	-	-	-	-	+	-	+	-	+

Extrolite produced (+); Extrolite not produced (-).

All the species of *Fusarium*, except *F. andiyazi*, produced fusarin C in this study. Beauvericin, bikaverin, deoxyfusapyron and fusapyron were found in cultures of certain species of *Fusarium*, *Neocosmospora* and *Neotestudina*. Specifically, *F. madaense* sp. nov. and *N. rosatii* produced all four aforementioned compounds together with the other three species of *Fusarium* for bikaverin and *F. andiyazi* for deoxyfusapyron and fusapyron. The immunosuppressant cyclosporins [A (mean: 42.2 mg/kg), B (mean: 27.3 mg/kg), C (mean: 29.9 mg/kg), D (mean: 4.6 mg/kg) and H (mean: 32.9 mg/kg)] were specific to *Neocosmospora* and were found only in cultures of *N. falciformis* and *N. vasinfecta*. Radicicol (323 mg/kg) and sulochrin (1.8 mg/kg) were found in one strain of *N. vasinfecta* and *N. rosatii*, respectively. Based on the recorded chemical profiles in this study, the three studied genera are closely related chemotaxonomically. However, *F. madaense* sp. nov. and *N. rosatii* seem to be more closely related than the other species. This is the first chemotaxonomic profiling of *F. madaense* sp. nov., *Meyerozyma*, *Neocosmospora* and *Neotestudina*.

Phylogenetic analyses of *Fusarium* and *Neocosmospora* and description of a novel *Fusarium* species

**Figure 3.**
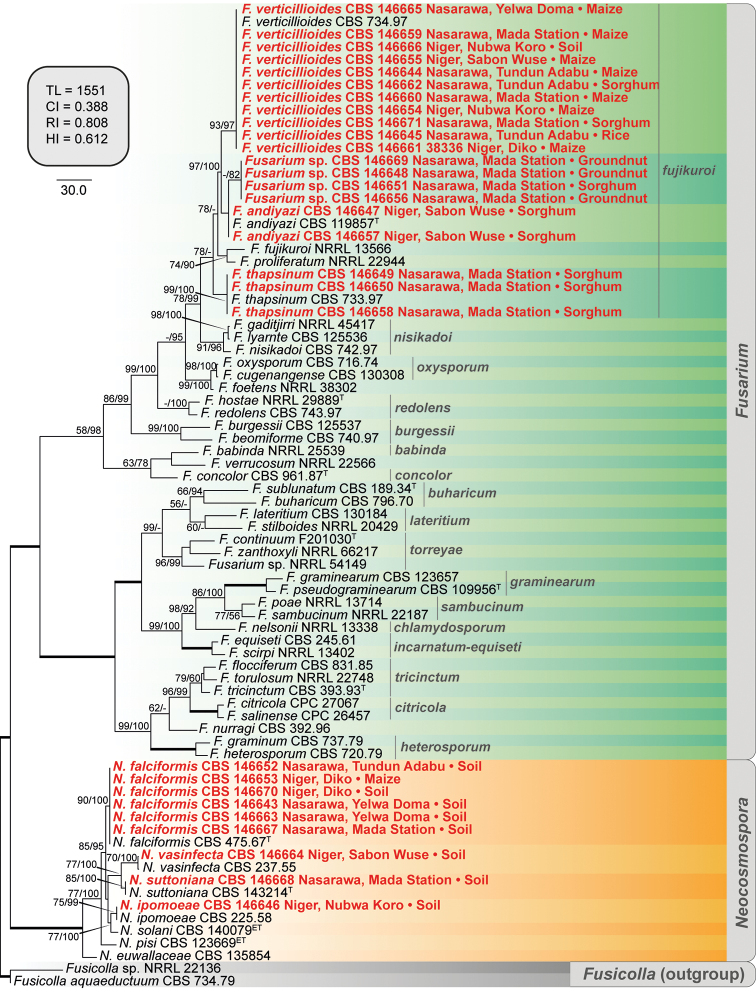
The first of 1000 equally parsimonious trees obtained from Maximum-Parsimony (MP) analysis of *RPB2* sequences of 76 isolates of *Fusarium* and *Neocosmospora* spp. Numbers on the nodes are MP bootstrap values (BS) and Maximum-Likelihood BS values above 70%. Branch lengths are proportional to distance. Ex-type and ex-epitype strains are indicated with ^T^ and ^ET^, respectively. The names of 17 species complexes of *Fusarium* are shown in grey. Nigerian isolates obtained in this study are shown in red together with their geographical origin and source of isolation. The internal square shows MP statistics as follows: TL = tree length, CI = consistency index, RI = retention index and HI = homoplasy index.

A first phylogenetic analysis, based on partial *RPB2* sequences, was conducted to identify Nigerian isolates morphologically compatible with *Fusarium* and *Neocosmospora* spp. (Fig. 3). The analysis included 659 positions of 78 isolates, including the two outgroup taxa (*Fusicolla aquaeductuum* NRRL 20686 and *Fusicolla* sp. NRRL 22136), of which 284 bp were constant sites, 375 bp were variable and 336 bp were parsimony-informative. The ingroup taxa included representative isolates of 40 species from 17 species complexes of *Fusarium* and seven species of *Neocosmospora*. Four isolates (CBS 146648, 146651, 146656 and 146669) clustered in a partially supported, putative novel clade, closely related to *F. andiyazi*; the latter taxon, however, clustered in an unresolved phylogenetic position.

**Figure 4.**
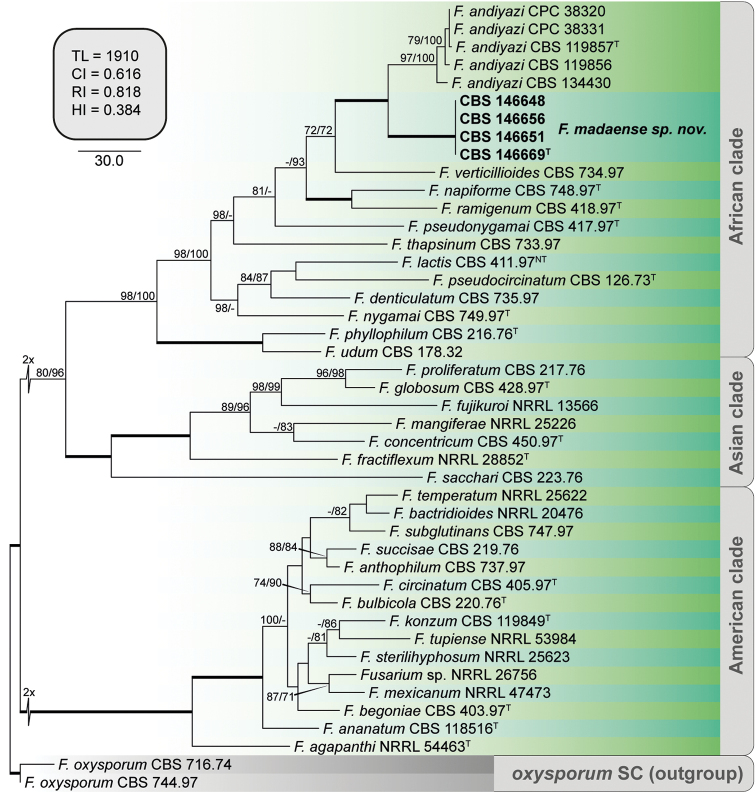
The first of 24 equally parsimonious trees obtained from Maximum-Parsimony (MP) analysis of *BenA, CaM, RPB1, RPB2* and *TEF-1a* sequences of 42 isolates of *Fusarium* spp. Numbers on the nodes are MP bootstrap values (BS) and Maximum-Likelihood BS values above 70%. Branch lengths are proportional to distance. Ex-type strains are indicated with ^T^. Strains corresponding to new species described here are shown in **bold**. The internal square shows MP statistics as follows: TL = tree length, CI = consistency index, RI = retention index and HI = homoplasy index.

To further determine the relationship between the putative novel clade and *F. andiyazi*, a second analysis was conducted which encompassed 4456 positions of five loci (*BenA* 525 bp, *CaM* 545 bp, *>RPB1* 978 bp, *RPB2* 1 735 bp and *TEF-1a* 673 bp), of which 3417 were constant (*BenA* 406 bp, *CaM* 421 bp, *>RPB1* 777 bp, *RPB2* 1 379 bp and *TEF-1a* 434 bp), 1018 were variable (*BenA* 118 bp, *CaM* 120 bp, *RPB1* 201 bp, *RPB2* 356 bp and *TEF-1a* 223 bp) and 614 were parsimony informative (*BenA* 64 bp, *CaM* 63 bp, *RPB1* 132 bp, *RPB2* 236 bp and *TEF-1a* 119 bp). The final alignment included 44 isolates, representing 35 *Fusarium* spp. from the three biogeographical phylogenetic clades of FFSC (African, American and Asian clades, O’Donnell et al. 1998) plus two outgroups (*F. oxysporum* NRRL 20433 and NRRL 22902) (Fig. 4). The multi-locus phylogeny confirmed the previous results. The putative novel clade (CBS 146648, 146651, 146656 and 146669) was resolved as a fully supported phylogenetic lineage (MPBS = 100, MLBS = 100), sister to a moderately-supported clade (MPBS 97, MLBS 100), encompassing the ex-type strain of *F. andiyazi* (CBS 119857), plus four additional representative isolates of the latter species, two of them (CBS 146647 and 146657) being obtained in this study. The novel phylogenetic lineage is here recognised as *Fusarium madaense* sp. nov.

### Taxonomy

#### Fusarium madaense

Taxon classificationFungiHypocrealesNectriaceae

Ezekiel, Sand.-Den., Houbraken & Crous sp. nov.

0AF4E7D6-D928-5D66-8DBA-28F37FA41AD5

835266

Figure 5

##### Diagnosis.

Different from *F. thapsinum* by the absence of napiform microconidia. Different from *F. andiyazi*, *F. thapsinum* and *F. verticillioides* by its lighter colony pigmentation, growth rates, microconidial septation, presence of true chlamydospores and secondary metabolite patterns.

##### Type.

Nigeria, Nasarawa, Mada Station, from groundnut (*Arachis hypogaea*), Sep. 2018, C.N. Ezekiel, holotype CBS H-24346, ex-holotype strain CBS 146669 = CPC 38344 = 12B(3)2.

**Figure 5.**
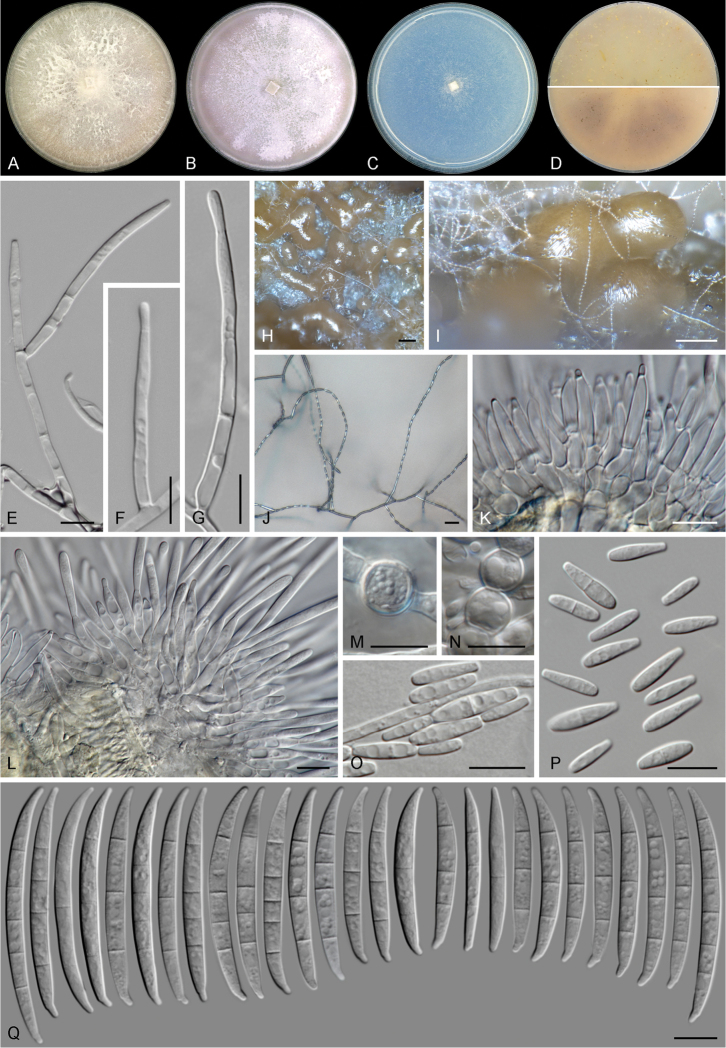
*Fusarium madaense* sp. nov. (ex-type culture CBS 146669). **A–C** aspect of colonies on PDA, OA and SNA, respectively, after 14 d at 24°C in the dark **D** colony reverse on OA (up) and PDA (down) after 14 d at 24 °C in the dark **E–G, J** aerial conidiophores and phialides **H, I** sporodochia formed on the surface of carnation leaves **K, L** sporodochial conidiophores **M, N** chlamydospores **O, P** microconidia **Q** sporodochial conidia. Scale bars: 100 μm (**H, I**); 20 μm (**J**); 10 μm (all others).

##### Description.

Colonies grown in the dark at 24°C. On MEA and PDA with an average radial growth rate of 5.9–6.5 mm/d and filling an entire 90 mm Petri dish in 7 d. Surface white to pale rosy buff, flat, velvety to felty with abundant patches of white aerial mycelium; margin regular, filiform. Reverse pale saffron to peach, a pale bay diffusible pigment can be scarcely produced. On OA, occupying an entire 90 mm Petri dish in 7 d. Surface white to pale rosy buff, flat, velvety to felty with abundant patches of white aerial mycelium; margin regular. Reverse pale luteous to saffron. On SNA, reaching 24–25 mm diam. in 7 d. Surface white, velvety, with scarce aerial mycelium, margins filiform. Reverse white.

*Conidiophores* on aerial mycelium straight, erect, septate, smooth- and thin-walled, commonly simple or reduced to conidiogenous cells, borne laterally on hyphae or laterally branched at various levels, bearing terminal single monophialides; *phialides* subulate to subcylindrical, smooth- and thin-walled, (17–)25.5–39.5 μm long, (2–)2.5–3.5 μm at widest point, periclinal thickening and collarettes inconspicuous or absent; *microconidia* hyaline, clavate, smooth- and thin-walled, 0–3-septate, (7–)9–15(–21) × (2–)2.5–4(–5) μm, arranged in long chains at the tip of monophialides. *Sporodochia* pale to bright orange, formed abundantly on the surface of carnation leaves and on agar surface. *Conidiophores* in sporodochia, 21–60 μm tall, simple or irregularly and verticillately branched, bearing terminal, single monophialides or groups up 2–3 monophialides; *sporodochial phialides* doliiform to subcylindrical, (10.5–)13–18(–20.5) × (2.5–)3–4(–4.5) μm, smooth- and thin-walled, with conspicuous periclinal thickening and an often short apical collarette. *Sporodochial conidia* lunate to falcate, tapering towards apical and basal ends, moderately curved dorsiventrally or with an almost straight ventral part; apical cell more or less equally sized than the adjacent cell, apically slightly elongated to papillate; basal cell distinctly notched, (0–)1–5(–6)-septate, hyaline, thin- and smooth-walled. Aseptate conidia: (38–)38.5–42(–44) × 3.5–4.5 μm; one-septate conidia: (37.5–)40–48(–53) × 3.5–4(–4.5) μm; two-septate conidia: 43 × 3.7 μm; three-septate conidia: (29–)38–48.5(–61.5) × (3–)4–4.5(–5) μm; four-septate conidia: (45–)46.5–54(–59) × (3.5–)4–4.5(–5) μm; five-septate conidia: 47.5–55.5(–60) × 4–4.5 μm; six-septate conidia: 55.5 × 4.5 μm; overall (29–)38.5–50(–61.5) × (3–)4–4.5(–5) μm. *Chlamydospores* present on MEA, PDA and SNA, globose to subglobose, hyaline, smooth and thick-walled, (6–)6.5–8.5(–10) μm diam., terminal or intercalary in the aerial hyphae, solitary or in chains

##### Distribution.

Nigeria.

##### Etymology.

Name refers to Mada Station, a locality in Nasarawa State, Nigeria, where the species was found.

##### Additional isolates examined.

Nigeria, Mada Station, from groundnut (*Arachis hypogaea*), Sept 2018, C.N. Ezekiel, CBS 146648 = CPC 38321 = 12B(3), CBS 146656 = CPC 38330 = 12B(5); from sorghum, Jan 2019, C.N. Ezekiel, CBS 146651 = CPC 38324 = 7S(6).

##### Notes.

Although clearly recognisable based on genetic markers, *Fusarium madaense* is hardly distinguishable from its closer relatives, based on morphological features only. The novel species is characterised by abundant long, slender and slightly curved macroconidia, a morphology typical of the FFSC. The overall morphology of *F. madaense* is similar to that of *F. andiyazi*, *F. thapsinum* and *F. verticillioides*; all species are characterised by clavate microconidia formed in long chains from relatively long monophialides. Moreover, the four mentioned species are known to be pathogenic on sorghum (Leslie and Summerell 2006) and have been isolated here from the same geographical regions. Nevertheless, some morphological features of *F. madaense* can provide an indication of its identity. These include a pale saffron colony pigmentation on OA and PDA, not developing the intense purple colour typical of *F. andiyazi* and *F. verticillioides*, nor the yellow pigmentation of *F. thapsinum*; the presence of up to 3-septate clavate microconidia vs. the up to 2-septate and aseptate microconidia of *F. andiyazi* and *F. verticillioides*, respectively; and the aseptate, but also rarely napiform microconidia of *F. thapsinum* (Nirenberg 1976, Klittich et al. 1997, Marasas et al. 2001). In addition, *F. madaense* can be differentiated from *F. andiyazi*, its closest morphological and phylogenetic relative, by its slightly faster growth rates on PDA, somewhat wider macroconidia and the presence of true chlamydospores.

The proposal of the novel species *F. madaense* and its differentiation from *F. andiyazi*, *F. thapsinum* and *F. verticillioides* is also supported by secondary metabolite profiling of all the above-mentioned species, as found in this study. *Fusarium madaense* was the only beauvericin-producing species in our dataset. Nevertheless, it has been reported that *F. verticillioides* strains can produce trace levels of this toxin (Leslie et al. 2004, Leslie and Summerell 2006). The alpha-pyrones deoxyfusapyron and fusapyron were produced only by *F. madaense* and its closest relative *F. andiyazi*; by contrast, fusarin C was produced by *F. madaense*, *F. thapsinum* and *F. verticillioides*, but not by *F. andiyazi*.

## Conclusions

We have shown the importance of applying robust taxonomic approaches to fungal characterisation in this study. Here, diverse fungal species, including those not previously reported from Nigerian food and soil, as well as a novel *Fusarium* species, *F. madaense* sp. nov., were identified and described. Several of these species possess mycotoxigenic, as well as plant, human and animal pathogenic, potential. We further elucidated the secondary metabolite profiles of strains within the identified fungal species. A handful of small molecule compounds were found in the cultures of the strains, including several compounds not previously reported from some strains; a few could serve as species-specific chemotaxonomic markers. Overall, we provide snapshot data on the fungal biodiversity in two north-central Nigerian states. The findings of this study are valuable to guide researchers to predict mycotoxin contamination of crops/food and possible sources of fungal infections in humans and animals, as well as to find, where unavailable and implement where available, strategies towards the control of problematic fungi and the adverse effects they may pose.

## Supplementary Material

XML Treatment for Fusarium madaense

## Supplementary materials

Supplementary material 1 Tables S1, S2 Data type COL Explanation note **Table S1.** Strain details and GenBank accession numbers of molecularly identified isolates included in this study. **Table S2.** Strain data and accession numbers of additional strains included in phylogenetic analyses of *Fusarium* and related taxa. https://binary.pensoft.net/file/419486This dataset is made available under the Open Database License (http://opendatacommons.org/licenses/odbl/1.0/). The Open Database License (ODbL) is a license agreement intended to allow users to freely share, modify, and use this Dataset while maintaining this same freedom for others, provided that the original source and author(s) are credited.Chibundu N. Ezekiel, Bart Kraak, Marcelo Sandoval-Denis, Michael Sulyok, Oluwawapelumi A. Oyedele, Kolawole I. Ayeni, Oluwadamilola M. Makinde, Oluwatosin M. Akinyemi, Rudolf Krska, Pedro W. Crous, Jos Houbraken
